# Market Impact of Foot-and-Mouth Disease Control Strategies: A UK Case Study

**DOI:** 10.3389/fvets.2017.00129

**Published:** 2017-09-01

**Authors:** Siyi Feng, Myles Patton, John Davis

**Affiliations:** ^1^Agri-Food and Biosciences Institute, Belfast, United Kingdom

**Keywords:** foot-and-mouth disease, partial equilibrium model, disease control strategy, market impact, economics

## Abstract

Foot-and-mouth disease (FMD) poses a serious threat to the agricultural sector due to its highly contagious nature. Outbreaks of FMD can lead to substantial disruptions to livestock markets due to loss of production and access to international markets. In a previously FMD-free country, the use of vaccination to augment control of an FMD outbreak is increasingly being recognized as an alternative control strategy to direct slaughtering [stamping-out (SO)]. The choice of control strategy has implications on production, trade, and hence prices of the sector. Specific choice of eradication strategies depends on their costs and benefits. Economic impact assessments are often based on benefit–cost framework, which provide detailed information on the changes in profit for a farm or budget implications for a government (1). However, this framework cannot capture price effects caused by changes in production due to culling of animals; access to international markets; and consumers’ reaction. These three impacts combine to affect equilibrium within commodity markets (2). This paper provides assessment of sectoral level impacts of the eradication choices of FMD outbreaks, which are typically not available from benefit–cost framework, in the context of the UK. The FAPRI-UK model, a partial equilibrium model of the agricultural sector, is utilized to investigate market outcomes of different control strategies (namely SO and vaccinate-to-die) in the case of FMD outbreaks. The outputs from the simulations of the EXODIS epidemiological model (number of animals culled/vaccinated and duration of outbreak) are used as inputs within the economic model to capture the overall price impact of the animal destruction, export ban, and consumers’ response.

## Introduction

Foot-and-mouth disease (FMD) poses a serious threat to the agricultural sector due to its highly contagious nature, which can lead to substantial disruptions to livestock markets. It is estimated that the outbreak in the UK in 2001 resulted in losses to agriculture and the food chain of approximately £3.1 billion, with further significant impacts on the wider economy (3). A *stamping-out (SO)* policy was implemented in 2001 to control the disease, whereby all infected stock and others exposed to infection (dangerous contact herds) were culled. Subsequent legislation included provisions for emergency vaccination as a control strategy. Vaccination was considered as an alternative control strategy during the 2007 outbreak, but ultimately was not deployed due to advice on the degree of risk of the disease spreading (4). There are two main vaccination strategies: *vaccinate-to-die (V-t-D)* and *vaccinate-to-live*. Under the *V-t-D* strategy, vaccinated animals are culled. Compared to the *SO* strategy, this may entail higher compensation spending, but shorten the duration of the outbreak by slowing the spread of the disease. Under the alternative *vaccinate-to-live* strategy, vaccinated animals remain in the population and may be slaughtered commercially, but this strategy entails delays in the reopening of international markets.

The market implications will vary across different control strategies due to, for example, differences in the duration of the outbreak, number of animals culled and closure of export markets. It is not straightforward to discern the market impact from previous outbreaks due to evolvement of contingency plans, country variations, dependency on export markets, other shocks to the market, etc. For example, when Argentina used vaccination to eradicate FMD in the early 2000s, the economy experienced severe downturns during this period, making it difficult to isolate the market impacts of the control strategy. FMD outbreaks are rare in countries, such as the UK, but will potentially have serious consequences if it occurs. It is, therefore, important to gain insights on disease management from not only historic experiences but also modeling exercises, which are built based on the logical abstraction of reality.

This paper investigates the market impacts of the strategies of *SO* versus *V-t-D* in FMD control in the case of the UK using a partial equilibrium modeling framework. The results are based on linking the FAPRI-UK partial equilibrium model and the EXODIS epidemiological model. Outputs from the simulations of the EXODIS model (number of animals culled/vaccinated and duration of outbreak) are used as inputs within the FAPRI-UK model to capture the price impact of the destruction of animals and restrictions to internal trade. The EXODIS model is stochastic, that is, the same virus can potentially result in a small- or a large-scale outbreak. Within this paper, market impacts are assessed for different scales of a potential outbreak. We begin with a review of the literature in Section “Literature Review.” This is followed by descriptions of the economic partial equilibrium model, the FAPRI-UK model and alternative scenarios in Section “Model and Scenarios.” The results are presented in Section “Results” and conclusions are drawn in Section “Summary and Discussion.”

## Literature Review

Due to the potential loss caused by FMD outbreaks, control strategies are constantly reviewed and evaluated, among which economic assessments are important. Economic assessments mostly concern the costs of alternative control strategies and/or value of certain responses, such as early detection, which help to reduce costs [e.g., Ref. (5, 6)]. These analyses are often based on the benefit–cost framework, which provide detailed information on the changes in profit for a farm or budget implications for a government (1). However, the benefit–cost framework cannot capture market price effects caused by changes in the following:

- Production due to culling of animals;
- Access to international markets; and
- Consumers’ reaction.

These three impacts combine to affect equilibrium within commodity markets (2). Reduced production as a result of the destruction of animals exerts a positive impact on price. Counteracting this, if exports are banned in response to the outbreak, additional production must be absorbed within the domestic market leading to an increase in supply. In addition, although FMD does not typically affect humans, there may be a negative consumption response to an outbreak due to consumer health concerns, even if these concerns are unfounded. Such concerns would lead to an inward shift in the demand curve and exert a downward impact on price. The ultimate impact on price depends on the weight of these individual effects and will vary across sectors depending on, for example, the importance of exports relative to domestic consumption. The partial equilibrium modeling framework models both the supply side and the demand side of a market and solves for a market clearance price. Thus, it is better suited to capture these effects. It enhances understanding of the market consequences for different commodities of different control strategies in response to an outbreak, complementing the benefit–cost analysis. There are assessments of FMD outbreaks and/or FMD control strategies using partial equilibrium models for the United States (7–10), Australia (11), Canada (12), and Mexico (13).

The economic impacts of vaccination as a control strategy are explicitly examined by Hagerman et al. (8), Schroeder et al. (9), Buetre et al. (11) and Tozer et al. (12), which reflect the rising recognition of this strategy in recent years. Both Hagerman et al. (8) and Buetre et al. (11) find that the desirability of vaccination depends on the scale of the outbreak. The cost of vaccination strategy cannot be justified when the outbreak is small. Also in the context of the US, Schroeder et al. (9) examines outbreaks at a larger scale compared to those in Hagerman et al. (8) and finds substantial benefits of using the vaccination strategy. Tozer et al. (12) is less informative in control strategy choices as it focuses on the dynamics of producer decisions using a discrete time optimal control model. The model assumes deterministic parameters that characterize the way in which FMD develops; in other words, there is no uncertainty with regard to the spread of the disease itself. To our knowledge, the market impacts of vaccination strategy for FMD control have not been examined in the UK. Following Hagerman et al. (8) and Buetre et al. (11), the control strategies will be assessed for potential outbreaks of different scales.

## Model and Scenarios

### Model

The FAPRI-UK model is an annual partial equilibrium model of the agricultural sector of the UK. Commodities modeled include wheat, barley, rapeseed, oats, beef, lamb, pork, poultry, dairy, and biofuel. Final demand for the meats and dairy entail derived demand for animals for slaughter and dairy cows and derived demand for feed from the crop sector. The dynamics in breeding herd building and livestock production are captured through appropriate lags in the equations. Production of these commodities is modeled at the level of the four countries of the UK: England, Wales, Scotland, and Northern Ireland. Demand is modeled at the UK level. Under most analyses, the model is run in conjunction with the EU-GOLD model so that markets clear at the EU level as markets of the Member States within the EU are deeply integrated.^1^^,^^2^ This means that international trade, excess supply, and demand at the UK level feed into the EU for solving the equilibrium prices.

The FAPRI-UK modeling system produces Baseline projections over a 10-year period of key variables in the beef, sheep, pig, poultry, dairy, and crop sectors for each country in the UK under the assumption that current policies remain in place and specific macroeconomic assumptions hold. The Baseline provides a benchmark against which projections of the policy scenarios can be compared and interpreted (14).^3^ The Baseline used in this analysis was finalized in Spring 2016 and covers the projection period 2016–2025.

When an FMD outbreak occurs, export of animal products from the outbreak country will be banned until the disease is eradicated and a specified waiting period has passed. During this period, the UK markets will be temporarily disintegrated from the EU. New equations for import and export of beef, lamb, and pork are developed so that these markets clear at the UK level. Then the export ban is incorporated as a shock to the export equation. The size of the shock depends on the duration of the disease outbreak and the waiting period. The time taken to eradicating the disease obviously depends on the success of the control strategy used, while the waiting period also depends on the control strategy as specified in existing regulations. Details of the waiting period for each of the control strategies examined within this paper and the specification of the size of the shock to export are provided in the next section.

In addition, the FMD outbreak will cause a shock to the production of the meat and dairy products as infected (and perhaps vaccinated livestock) are culled. Given the biological dynamics in livestock sector, culled livestock have impacts on meat production beyond the outbreak year, particularly in the beef sector. In general, if commodities redirected from export outweigh the reduction in production following an FMD outbreak, this results in excess supply, which exerts a downward impact on price in the domestic market. Price falls may deepen, depending on whether the outbreak causes a food scare in consumption.^4^

The last route through which equilibrium is restored is import adjustment. Imports will reduce in response to lower prices in the UK as exports being redirected to the domestic market. Nevertheless, domestic prices would rarely be higher than EU prices during the year of outbreak as imports are always possible. For the markets to reach equilibrium following an FMD outbreak, the price elasticity of import is a particularly crucial parameter; that is, the extent of import changes relative to price change. It is important to acknowledge that there is considerable uncertainty regarding the extent to which imports are likely to be displaced by the rechanneling of exports to the domestic market. The rate of displacement is of particular concern as the export ban implies a sudden substantial increase in supply to the domestic market from the rechanneled exports. This has important implications on the price impact of an FMD outbreak. Imports may be slow to readjust due to contractual reasons and demand requirements, e.g., imports from the southern hemisphere may fulfill demand requirements during specific periods of the season. It is also possible that imports adjust quickly in response to the rechanneling of exports. As a result, sensitivity analyses regarding import adjustments are carried out in which changes in imports are exogenously imposed. Two extreme cases are examined: no displacement and substantial displacement. In the case of no displacement, it is assumed that imports remain unchanged compared to Baseline projections. This reflects the assumption that imports are slow to adjust and cannot be readily canceled. In the case of substantial displacement, it is assumed that imports are reduced by 90% of exports that are diverted to the domestic market due to the export ban, implying that imports adjust instantaneously in response to the imposition of an export ban. The sensitivity analysis provides a means to quantify the price impact of an FMD outbreak under different trade assumptions.

### Scenarios

Two FMD control strategies are examined in this paper.

#### Stamping-Out

Under this scenario, numbers of animals culled from simulations of the epidemiological model are incorporated within the economic model, resulting in reductions in livestock numbers and animals available for slaughter. In addition to the number of culled animals, the epidemiological model provides data on the duration of the outbreak. Under the “SO” scenario, the waiting period for applying for disease-free status and resuming export is 90 days after the last case of FMD.

#### Vaccinate-to-Die

Similar to scenario (i), numbers of culled animals from the epidemiological model are entered as supply shocks in the economic model and exports resume 90 days after the last infected and vaccinated animals are culled.

The analysis undertaken in this paper is based on stochastic simulations of the EXODIS epidemiological model undertaken by the Animal and Plant Health Agency as an extension of Exercise Rowan.^5^ The epidemiological model simulations are based on an outbreak equivalent to the characteristics of the virus in the UK in 2001, but take into account up-to-date UK contingency plans. The stochastic output from the epidemiological model yielded 200 outcomes, which reflect alternative developments of the same FMD virus. In order to identify the market impact of these different outcomes, the median outputs from the epidemiological model are used as inputs within the economic model. In addition, we consider the tails of the distribution from the epidemiological model outcomes. In particular, the 95th percentile is used to represent the situation in which the virus develops into a particularly serious outbreak, as reflected in the number of livestock affected and the duration of the disease outbreak. The 5th percentile replicates a mild outbreak. As shown in the summary statistics in Table 1, the number of culled animals is higher under the V-t-D simulations compared to SO. However, the duration of the disease-free status period is lower under the former, particularly with regard to the 95th percentile. A matching procedure was used to identify individual simulations that approximate the median, 5th and 95th percentile statistics of all the relevant variables (numbers of livestock culled of different species and duration of disease outbreak).^6^

**Table 1:** Summary statistics from EXODIS epidemiology model.

		Stamping-out	Vaccinate-to-die
Infected premises	Median	230	120
	5 Percentile	134	75
	95 Percentile	360	181
Period to apply for disease-free status (days)	Median	171	141
	5 Percentile	152	129
	95 Percentile	224	176
Total culled animals	Median	342,558	1,020,682
	5 Percentile	191,310	636,701
	95 Percentile	593,892	1,444,701
Total vaccinated animals	Median	–	837,518
	5 Percentile	–	529,050
	95 Percentile	–	1,174,954

Animals culled under the *SO* scenario in the simulated outbreak of median scale (based on the epidemiological model) represent 1.8, 0.6, and 0.2% of the projected total number slaughtered of the year for the beef, sheep, and pig sector, respectively. The percentages rise to 4.8, 1.9, and 0.7% in the *V-t-D* scenario. However, it should be noted that while the breeding herd and animals at different life stages are not distinguished in the epidemiological model, they are modeled separately in the economic model. Therefore, total number culled by species are proportioned to the breeding herd and animal for meat purpose at different life stages based on historic census before they are incorporated into the economic model. This implies that the percentages mentioned earlier are greater than the production shock to the year of the outbreak while the culling will exert some effects for the year(s) following the outbreak.

The FMD outbreak in the UK in 2001 lasted for 221 days and the number of animals culled was over 4 million. By contrast, the 2007 outbreak lasted for 58 days and only 2,160 animals were culled (4). It appears that the 2001 outbreak is more serious than the case of 95th percentile presented in this study (Table 1). Although the epidemiological simulations are based on an outbreak equivalent to the characteristics of the virus in the UK in 2001, direct comparison is difficult because the model simulations take into account the up-to-date UK contingency plans, which have been significantly reviewed and updated following the outbreaks.

Underlying the *SO* and *V-t-D* scenarios, it is assumed that all exports of beef, sheep, and pig meat are halted for the duration of the outbreak plus 3 months after the detection of the last case, in line with World Animal Health Guidelines. Thus, it is assumed that there is no regionalization, i.e., exports from the whole country are banned. The reduction in exports as a result of the export ban is computed as a proportion of the length of the export ban:

Export Reduction=Export under the Baseline×(Days of Export Ban/365).

The length of the export ban is defined as the period of the last reported case plus the waiting period before it is possible to apply for disease-free status. This definition may be interpreted as the most optimistic estimation of the duration of the export ban as it implies no delay in approval. In the past two FMD outbreaks in the UK (2001 and 2007), both outbreaks ended in around September of the year and the UK regained disease-free status in the beginning of the following year. This suggests that the waiting period in these two cases were not much longer than the minimum required (i.e., 3 months). However, it should be noted that in both cases, the UK used the *SO* strategy only. A summary of the scenarios (including sensitivity analysis of import adjustments) is presented in Table 2.

**Table 2:** Foot-and-mouth disease (FMD) control scenarios.

FMD control strategies	Percentiles	Export ban period (days)	Trade assumptions		
*Stamping-Out*	5th	152	*Endogenous displacement*	*No displacement*	*Substantial displacement*
Export ban = disease period + 90 days	50th (median)	171	Imports are partially displaced by absorption of exports on the domestic market depending on changes in relative price	Imports remain unchanged	Imports reduced by 90% of exports that are absorbed on the domestic market
	95th	224			
*Vaccinate-to-die*	5th	129			
Export ban = disease period + 90 days	50th (median)	141			
	95th	176			

## Results

### Impact during the Year of Outbreak

Table 3 reports the impacts of the SO and V-t-D strategies for outbreaks of difference scales with various assumptions on import adjustment during the year of outbreak.

**Table 3:** Foot-and-mouth disease control strategy results—comparison between baseline projections and scenario in year of outbreak (2017).

	Baseline	Endogenous displacement						No displacement						Substantial displacement					
		Stamping-out			Vaccinate-to-die			Stamping-out			Vaccinate-to-die			Stamping-out			Vaccinate-to-die		
		5th	Median	95th	5th	Median	95th	5th	Median	95th	5th	Median	95th	5th	Median	95th	5th	Median	95th
**Beef sector**																			
Production (1,000 t)	906	904	903	897	901	898	895	904	903	897	901	898	895	904	903	897	901	898	895
Consumption (1,000 t)	1,107	1,139	1,143	1,151	1,132	1,133	1,138	1,159	1,165	1,178	1,147	1,149	1,158	1,111	1,110	1,106	1,106	1,104	1,102
Net exports (1,000 t)	−201	−235	−240	−254	−231	−235	−243	−255	−262	−281	−247	−251	−263	−206	−207	−209	−205	−206	−207
Price (£/100 kg dw)	318	295	293	288	300	300	296	285	282	276	293	292	286	316	317	322	320	321	322
Output (£ million)	2,881	2,668	2,645	2,587	2,705	2,691	2,648	2,581	2,549	2,481	2,639	2,620	2,561	2,857	2,858	2,885	2,878	2,880	2,881
*Changes in percent*																			
Production		−0.2%	−0.3%	−0.9%	−0.6%	−0.9%	−1.2%	−0.2%	−0.3%	−0.9%	−0.6%	−0.9%	−1.2%	−0.2%	−0.3%	−0.9%	−0.6%	−0.9%	−1.2%
Consumption		2.9%	3.2%	4.0%	2.2%	2.3%	2.9%	4.7%	5.2%	6.4%	3.7%	3.8%	4.7%	0.3%	0.3%	−0.1%	−0.1%	−0.2%	−0.4%
Price		−7.2%	−7.9%	−9.3%	−5.5%	−5.8%	−7.0%	−10.2%	−11.2%	−13.1%	−7.8%	−8.3%	−10.0%	−0.6%	−0.4%	1.1%	0.5%	0.8%	1.3%
Output		−7.4%	−8.2%	−10.2%	−6.1%	−6.6%	−8.1%	−10.4%	−11.5%	−13.9%	−8.4%	−9.0%	−11.1%	−0.8%	−0.8%	0.2%	−0.1%	0.0%	0.0%
**Sheep sector**																			
Production (1,000 t)	319	319	318	316	316	313	310	319	318	316	316	313	310	319	318	316	316	313	310
Consumption (1,000 t)	313	338	341	350	332	333	337	364	369	386	353	355	363	317	317	318	314	312	309
Net exports (1,000 t)	7	−19	−23	−33	−16	−19	−27	−45	−52	−70	−37	−41	−53	1	1	−1	2	2	1
Price (£/100 kg dw)	375	291	283	257	307	306	292	224	213	182	247	244	225	358	359	354	370	380	389
Output (£ million)	1,199	927	898	815	971	957	906	715	677	577	782	766	698	1,140	1,140	1,121	1,170	1,192	1,207
*Changes in percent*																			
Production		−0.3%	−0.6%	−1.0%	−1.1%	−1.9%	−3.0%	−0.3%	−0.6%	−1.0%	−1.1%	−1.9%	−3.0%	−0.3%	−0.6%	−1.0%	−1.1%	−1.9%	−3.0%
Consumption		7.9%	8.8%	11.8%	6.1%	6.3%	7.7%	16.3%	18.0%	23.4%	12.9%	13.4%	16.1%	1.4%	1.3%	1.5%	0.3%	−0.4%	−1.1%
Price		−22.5%	−24.7%	−31.4%	−18.1%	−18.6%	−22.1%	−40.2%	−43.2%	−51.4%	−34.1%	−34.9%	−40.0%	−4.7%	−4.4%	−5.6%	−1.3%	1.3%	3.7%
Output		−22.7%	−25.1%	−32.1%	−19.0%	−20.2%	−24.4%	−40.4%	−43.5%	−51.9%	−34.8%	−36.1%	−41.8%	−4.9%	−4.9%	−6.5%	−2.4%	−0.6%	0.6%
**Pig sector**																			
Production (1,000 t)	916	911	910	908	909	905	902	910	909	907	908	905	901	913	913	912	911	908	905
Consumption (1,000 t)	1,429	1,501	1,510	1,536	1,488	1,491	1,506	1,520	1,531	1,563	1,504	1,507	1,526	1,436	1,437	1,439	1,433	1,430	1,429
Net exports (1,000 t)	−513	−590	−600	−628	−579	−586	−604	−610	−622	−656	−595	−603	−625	−523	−524	−527	−521	−522	−524
Price (£/100 kg dw)	132	112	109	103	115	114	110	106	103	95	110	109	104	130	130	129	131	132	132
Output (£ million)	1,211	1,017	995	935	1,046	1,035	996	963	936	866	999	986	940	1,186	1,183	1,172	1,194	1,198	1,197
*Changes in percent*																			
Production		−0.6%	−0.7%	−0.9%	−0.8%	−1.2%	−1.5%	−0.7%	−0.8%	−1.0%	−0.9%	−1.3%	−1.6%	−0.4%	−0.4%	−0.5%	−0.5%	−0.9%	−1.2%
Consumption		5.0%	5.7%	7.5%	4.1%	4.3%	5.4%	6.3%	7.1%	9.4%	5.2%	5.5%	6.8%	0.5%	0.5%	0.7%	0.2%	0.0%	0.0%
Price		−15.5%	−17.3%	−22.2%	−12.9%	−13.5%	−16.5%	−19.9%	−22.1%	−27.8%	−16.8%	−17.6%	−21.1%	−1.7%	−2.0%	−2.8%	−0.9%	−0.1%	0.1%
Output		−16.0%	−17.9%	−22.8%	−13.6%	−14.6%	−17.8%	−20.5%	−22.7%	−28.5%	−17.6%	−18.6%	−22.4%	−2.1%	−2.3%	−3.2%	−1.4%	−1.1%	−1.2%

Starting with the “*SO—endogenous displacement*” scenario, UK prices fall by 7.9, 24.7 and 17.3%, respectively, in the beef, sheep, and pig sectors in 2017 for an outbreak of median scale. The negative price impact is attributable to the additional production absorbed onto the domestic market due to the export ban, which leads to an increase in domestic supply. The limited decline in production is insufficient to offset the rechanneling of exports and, hence, commodity prices decline. The sheepmeat sector experiences the greatest price decline due to the high level of self-sufficiency. The projected value of output in the sheepmeat sector falls by 25.1% and primarily reflects the drop in price.

Compared to the median, the negative price impact is greater following a more serious outbreak (the 95th percentile version of the scenario). The prices of beef, sheep meat, and pig meat fall by 9.3, 31.4, and 22.2%, respectively. Despite the more serious nature of the outbreak, the prices are lower under the 95th simulations compared the median. The negative production impact from the larger number of animals culled is more than offset by the longer duration of the outbreak, which results in an extended export ban and more produce being absorbed on the domestic market. By contrast, the milder outbreak experienced under the 5th percentile version of the scenario results in smaller price impacts compared to the median.

Under the “*endogenous displacement*” simulations the absorption of exports onto the domestic market is partially counteracted by a fall in imports. When it is assumed that imports do not adjust to the export ban (“no displacement” scenario), price drops are more severe. In the case of the median outbreak, beef, sheep meat, and pig meat prices fall by 11.2, 43.2, and 22.1%, respectively. Within this scenario, the absorption of exports onto the domestic market is not counteracted by a fall in imports, leading to a greater negative supply shock. While this “*no displacement*” scenario is extreme it sheds light on situations in which imports adjust slowly to an export ban. It thereby provides an indication of the implications of this assumption.

The price impact is significantly less marked when imports almost fully readjust in response to the rechanneling of exports. Under the median version of the “*SO—substantial displacement*” in which it is assumed that imports are reduced by 90% of exports, the sheepmeat price is 4.4% lower than the Baseline. This contrasts with 43.2% in the no displacement scenario.

Compared to “*SO*”, “*V-t-D*” leads to the culling of more animals and, hence, lower production. In addition, the “*V-t-D*” control strategy also significantly curtails the time-span of the outbreak and, as a consequence, the duration of the export ban. As a result, fewer exports are absorbed onto the domestic market. As a consequence of both these effects, the price impacts are less marked under the “*V-t-D*” scenarios compared to “*SO*.” For example, the sheep meat price falls by 18.6% under the median “*V-t-D—endogenous displacement*” scenario, compared to 24.7% under the equivalent “*SO—endogenous displacement*” scenario. The projected value of output falls by a greater amount than price in percentage terms (20.2 versus 18.6%) due to the fall in production.

Comparing the results of the outbreaks at the same quantile under the *V-t-D* and *SO* scenarios, it is apparent that the projected price and value of output differences between these strategies are more marked for severe outbreaks. With regard to the sheepmeat price the difference between the two control strategies under the 95th percentile versions of these scenarios is 9.3% as the price fall under *V-t-D* is 22.1 and 31.4% under SO. This compares to 6.1% under the median. This result supports the hypothesis that the benefits of vaccination are clearer for more severe outbreaks.

Similar to the “*SO*” results, the price impacts are significantly greater when it is assumed that imports remain unchanged compared to the endogenous versions of the scenario, which entail partial adjustments in imports. For example, under the median version of the “*V-t-D—no displacement*” scenario, the sheepmeat price falls by 35%. Again, the benefits of vaccination are greater under the 95th percentile compared to the median.

**Figure 1:**
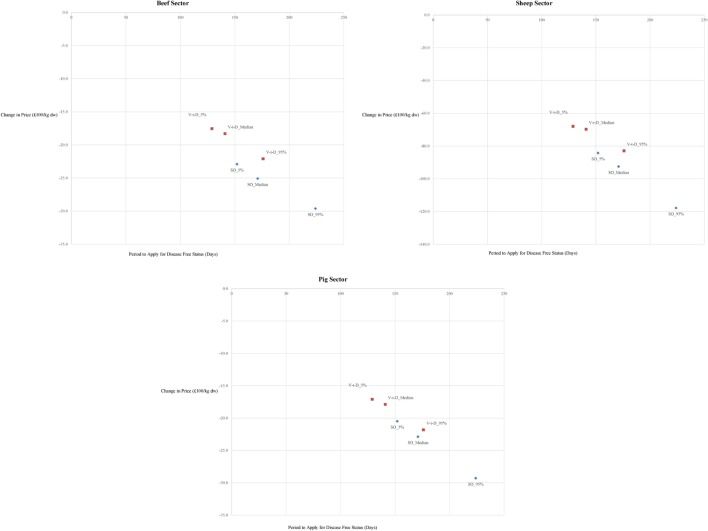
Changes in beef (upper left), sheepmeat (upper right), and pig prices (lower center) versus export ban period in the main “endogenous displacement” scenario (SO, stamping-out; V-t-D, vaccinate-to-die).

In general, across the scenarios the longer the duration of the export ban the greater the price fall (Figure 1). One exception is the “*V-t-D*” 95th percentile scenario. Under the “*V-t-D*” 95th percentile scenario, the export ban is 176 days, compared to 171 under the “*SO*” median scenario. Despite this, the price decline is less marked under the former. The number of livestock culled under the “*V-t-D*” 95th percentile is greater than the “SO” median case and, consequently, the rechanneling of exports to the domestic market leads to smaller excess supply.

### Impact over the Whole Projection Period

**Figure 2:**
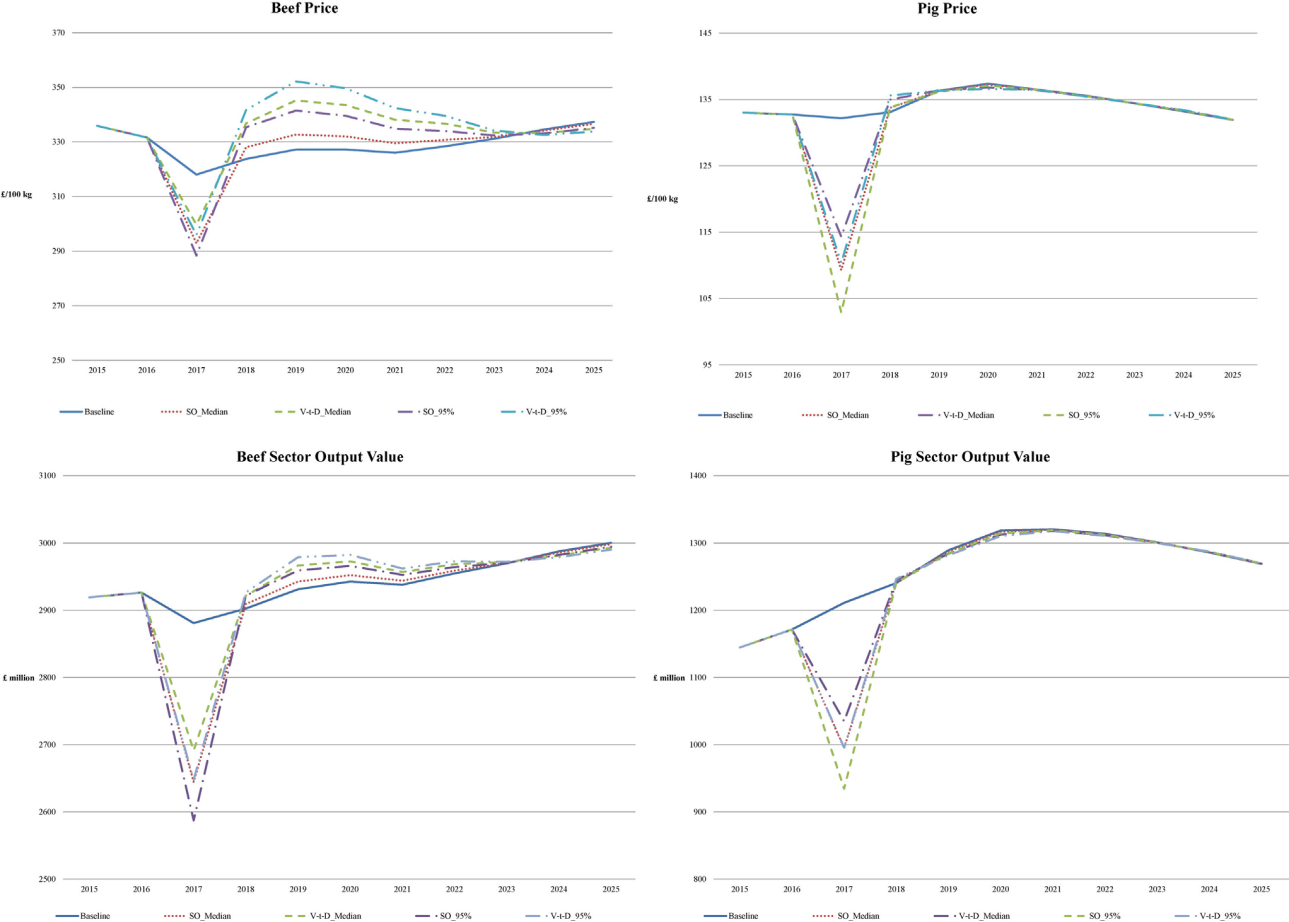
Price and output value paths of the beef (upper left, lower left) and pig (upper right, lower right) sectors 2015-2025 in the main “endogenous displacement” scenario (SO, stamping-out; V-t-D, vaccinate-to-die).

While commodity prices in the livestock sector are negatively affected by an FMD outbreak, this impact generally lasts for less than a year even under a serious outbreak (the longest among all the scenarios is 224 days in the case of the 95% percentile under the SO strategy). An outbreak will result in a smaller herd for the following year; breeding herd and animals for meat production numbers are smaller. To rebuild the breeding herd, some animals that would have been designated for meat production will be kept for breeding instead. As a result, meat production will be lower and prices will be higher compared to the Baseline. Among the three sectors, the restocking process is the longest for the beef sector and, therefore, beef price is the slowest to return to baseline level (Figure 2).^7^ Beef prices do not return to the baseline level until the year 2023, while in the pig sector there is no discernible impact from the year 2019 onward. The paths of output values, defined as production multiplied by price, are similar to the price paths, suggesting that the increases in prices outweigh the smaller production (Figure 2).

To further compare the impact of the disease on the output value of the livestock sectors, output values are summed over the period of 2017–2025. All values are discounted to the 2017 value before the summation is carried out, using a discount rate of 3.5%.^8^ Results are presented in Table 4. Over the period of 2017–2025, there are some small reductions in the total output values in the livestock sector; furthermore, the more severe the outbreak, the greater impact on total output value. Taking the longer term impact into account, the main conclusion from the previous section is still valid: the vaccination-to-die strategy helps to mitigate the market impact compared to the SO strategy.

**Table 4:** Change in total output values of the livestock sectors of 2017–2025.

	Baseline (£ million)	Stamping-out			Vaccinate-to-die		
		5th	Median	95th	5th	Median	95th
Cattle	23,059	−0.8%	−0.9%	−0.9%	−0.4%	−0.4%	−0.5%
Pig	10,045	−2.0%	−2.2%	−2.8%	−1.7%	−1.8%	−2.3%
Sheep	9,832	−2.8%	−3.1%	−4.0%	−2.4%	−2.6%	−3.2%
Total livestock	59,052	−1.1%	−1.2%	−1.5%	−0.8%	−0.9%	−1.1%

## Summary and Discussion

This study is made possible following a new component developed in the partial equilibrium model that enables the UK markets to deviate from the EU. In the two most recent FMD outbreaks in the UK (very serious in 2001 versus mild in 2007), emergency vaccination was never used. The effectiveness of vaccination is shown in the experiences of other countries (for example, the Netherlands and Uruguay). The Netherlands had used emergency vaccination combined with the *V-t-D* strategy in 2001. The culling of large number of vaccinated healthy animals was not without controversy. Since then, there is ongoing exploration of the *vaccinate-to-live* strategy. However, the use of *vaccinate-to-live* strategy entails a longer export ban, which raises concerns with the industry. There could also be other issues such as logistics. Therefore, better understanding of the trade-offs of the different strategies is needed to assist decision making, which is the main purpose of our study. In a future study, we will cover the strategy of *vaccinate-to-live*.

By combining epidemiology and partial equilibrium modeling frameworks the analysis undertaken in this study demonstrates the potential market consequences of alternative FMD control strategies. It is projected that an FMD outbreak has a negative impact on market prices and value of output, regardless of the control strategy. Although the analysis is based on a virus similar to the characteristics of the 2001 outbreak, unlike this previous outbreak, the number of animals culled and, hence, the production impact is relatively modest. This reflects the evolvement of contingency plans, with co-ordination measures helping to reduce the spread of disease. While the projected decline in production under both the *SO* and *V-t-D* scenarios results in lower value of output, the largest impact on value of output stems from the drop in price due to the closure of export markets. Similarly, studies in other geographical areas have shown that the export ban exerts the larger impact on farm revenue compared with production changes [e.g., Ref. (2, 7)].

The more severe the disease outbreak, the greater the negative price impact, as demonstrated by comparing the median and 95% percentile versions of the scenarios. While the latter results in the culling of more animals compared to the former, which exerts an upward impact on price, this is more than offset by the market impacts of the longer duration of the export ban.

It is important to acknowledge that underlying this analysis it is assumed that exports are halted for the full duration of the outbreak plus 90 days after the last case or the last vaccinated animal is culled. The price and value of output impact would be diminished if export markets were to reopen sooner. Potentially, governments could pursue regionalization, whereby trade is allowed to resume from non-infected regions, providing it is possible to demonstrate the disease is contained (2).

In addition, the feasibility of readjusting imports is crucial. The sensitivity scenarios indicate the extent to which readjustments in imports diminish the price and revenue impacts of an FMD outbreak. If it is not possible to reduce imports swiftly the price impact could be substantial, as demonstrated under the *no displacement* scenarios. This case represents the most marked potential impact. Exporters to the UK may choose to re-channel exports to other markets if prices were to decline significantly. However, the response is unlikely to be instantaneous.

Finally, the results of this analysis indicate that the price and value of output impacts are lower under *V-t-D* compared to *SO*. This conclusion holds when longer term impacts are taken into account. This primarily reflects the effectiveness of *V-t-D* in slowing the spread of the disease and, hence, curtailing the duration of the export ban. This comparison is based on the assumption that there are no delays in gaining the approval of reopening export markets. In reality, this may be more difficult with regard to vaccination due to logistical reasons, e.g., additional surveillance requirements for proof of freedom status and delays in removing vaccinated animals following the outbreak (15). The finding that vaccination is favored compared to SO is greater, the more severe the outbreak. However, it should be noted that this analysis focuses on the market impact of the disease outbreak. To make the final choice among the control strategies, other costs such as on farm and administrative costs should also be taken into account.

## Author Contributions

Substantial contributions to the conception or design of the work: SF, MP, and JD. Acquisition, analysis, or interpretation of data for the work: SF and MP. Drafting the work or revising it critically for important intellectual content: SF, MP, and JD. Final approval of the version to be published: SF, MP, and JD. Agreement to be accountable for all aspects of the work in ensuring the questions related to the accuracy or integrity of any part of the work are appropriately investigated and resolved: SF, MP, and JD.

## Conflict of Interest Statement

The authors declare that the research was conducted in the absence of any commercial or financial relationships that could be construed as a potential conflict of interest.

## Footnotes

^1^The EU-GOLD model is a partial equilibrium model of the agricultural sector at the EU level. It is developed and maintained by the Food and Agricultural Policy Research Institute (FAPRI) at the University of Missouri.

^2^This study was carried out before the UK referendum of exiting the EU.

^3^Project information on the AFBI website: https://www.afbini.gov.uk/analysis-agricultural-commodity-markets-fapri-uk-project.

^4^Note the analysis in this paper focuses on the effect of shock to production and exports. We do not examine the implication of a shock in consumption as it is difficult to discern the size of such a shock from real data as the scale of the shock in the simulations is smaller than the 2001 outbreak.

^5^The initial phase of Exercise Rowan was undertaken in the latter part of 2015. During the exercise, the EXODIS model was used to test FMD response capability in the UK. See Roche et al. (15) for further information on the EXODIS model.

^6^Rather than devising a rule to disaggregate UK epidemiology outputs at the regional level, individual simulations that approximate the median, 5th and 95th percentile statistics of all the relevant variables (numbers of livestock culled of different species and duration of disease outbreak) at the UK-level were identified. It is relatively straightforward to identify individual simulations that closely approximate the 5th percentile and median, but less so for the 95th percentile of all the sectors simultaneously. With regard to individual simulations, some sectors are not affected to the same extent compared to the statistics for the 95th percentile. More emphasis was placed on the beef and sheep sectors within the matching procedure. The relevant variables from the chosen individual simulations closely match the 95th statistics for these sectors, but slightly lower outcomes emerged for the pig sector. Nevertheless, the differences were sufficiently small such that qualitative differences between the scenarios for all the sectors, including the pig, still hold.

^7^Note: the intertemporal results discussed in this section draw on the main analysis, i.e., the “Endogenous Displacement” scenario.

^8^The choice of discount rate is based on Treasury Guidance of the UK government [the Greenbook by Treasury (16)].
